# Vitamin D status is heritable and under environment‐dependent selection in the wild

**DOI:** 10.1111/mec.16318

**Published:** 2022-01-07

**Authors:** Alexandra M. Sparks, Susan E. Johnston, Ian Handel, Jill G. Pilkington, Jacqueline Berry, Josephine M. Pemberton, Daniel H. Nussey, Richard J. Mellanby

**Affiliations:** ^1^ Institute of Evolutionary Biology School of Biological Sciences University of Edinburgh Edinburgh UK; ^2^ Faculty of Biological Sciences School of Biology University of Leeds Leeds UK; ^3^ Royal (Dick) School of Veterinary Studies and The Roslin Institute The University of Edinburgh Hospital for Small Animals Roslin UK; ^4^ Specialist Assay Laboratory (Vitamin D) Clinical Biochemistry Manchester Royal Infirmary Manchester UK

**Keywords:** 25 hydroxyvitamin D, fitness, GWAS, heritability, Soay sheep, vitamin D

## Abstract

Vitamin D has a well‐established role in skeletal health and is increasingly linked to chronic disease and mortality in humans and companion animals. Despite the clear significance of vitamin D for health and obvious implications for fitness under natural conditions, no longitudinal study has tested whether the circulating concentration of vitamin D is under natural selection in the wild. Here, we show that concentrations of dietary‐derived vitamin D_2_ and endogenously produced vitamin D_3_ metabolites are heritable and largely polygenic in a wild population of Soay sheep (*Ovis aries*). Vitamin D_2_ status was positively associated with female adult survival, and vitamin D_3_ status predicted female fecundity in particular, good environment years when sheep density and competition for resources was low. Our study provides evidence that vitamin D status has the potential to respond to selection, and also provides new insights into how vitamin D metabolism is associated with fitness in the wild.

## 1 INTRODUCTION

Vitamin D is a critical component in the development and maintenance of skeletal health (Elder & Bishop, 2014). However, current research suggests that vitamin D may also have wider biomedical effects, with studies linking vitamin D insufficiency to increased risk of mortality in humans and companion animals (Schöttker et al., 2014; Titmarsh, Gow, et al., 2015; Titmarsh, Kilpatrick, et al., 2015), as well as reproductive failure, low birth weight, infertility and reduced litter sizes in humans, rats and mice (Halloran & Deluca, 1980; Luk et al., 2012; Tous et al., 2019; Yoshizawa et al., 1997). This link suggests that vitamin D status may be associated with fitness in natural populations, yet there have been no investigations into the causes and consequences of its variation in wild populations. To understand the ecological and evolutionary significance of vitamin D metabolism, it is necessary to determine how it varies due to individual and environmental variation, the extent to which it is heritable and the underlying genetic architecture, and its association with key components of fitness, such as survival and fecundity. By investigating these factors, we can begin to understand the evolutionary potential of vitamin D status and determine if it is under selection in natural conditions.

The availability of vitamin D to mammals is dependent on the environment. Vitamin D_3_ can be produced in the skin of most mammals following exposure to sunlight, with notable exceptions including cats and dogs (How et al., 1994; Hurst, Homer, et al., 2020). Vitamin D_3_ can also be obtained from dietary sources, notably oily fish, eggs and liver, whereas vitamin D_2_ can only be obtained in the diet from some plants and fungi (Elder & Bishop, 2014). Human studies suggest vitamin D status, typically assessed by measuring circulating concentrations of the major metabolite 25 hydroxyvitamin D (25(OH)D), is heritable, with between 0% and 86% of variation due to genetic effects (Arguelles et al., 2009; Jiang et al., 2018; Karohl et al., 2010; Mills et al., 2015; Orton et al., 2008; Revez et al., 2020). Genome‐wide association studies (GWAS) have also identified several genes involved in relevant pathways which contribute significantly to this genetic variation (Ahn et al., 2010; Hiraki et al., 2013; Jiang et al., 2018; Manousaki et al., 2017, 2020; Revez et al., 2020; Wang et al., 2010). However, vitamin D supplementation and artificial exposure to ultraviolet light are increasingly common in Western human populations, whilst variation in challenging aspects of the environment (e.g., thermoregulation, infection, food limitation) are greatly reduced compared to most natural populations (Kupferschmidt, 2012). As a result, there is little known of the potential for genes identified in human studies to contribute to variation in vitamin D metabolism and thus potentially be under natural selection more broadly.

Here, we investigate the genetic architecture and fitness consequences of vitamin D status in a long‐term study of wild Soay sheep on the St Kilda archipelago in NW Scotland. A previous small‐scale study of females sampled in a single year showed that plasma 25(OH)D_2_ and 25(OH)D_3_ concentrations varied among individuals, and identified associations between vitamin D status, age, coat colour and female fecundity (Handel et al., 2016). In the present study, we use samples collected longitudinally over a 6‐year period, alongside detailed information on the genetics and life histories of the individuals sampled, to: (i) estimate the heritability of vitamin D status in the wild; (ii) use a GWAS approach to determine the genetic architecture of vitamin D status; and (iii) examine how vitamin D status predicts survival and reproductive performance.

## 2 METHODS

### 2.1 Study population

The Soay sheep is a primitive breed of domestic sheep that was isolated on the island of Soay in the remote St Kilda archipelago (57°49′N, 8°35′W) several millennia ago, and has been living wild since then (Clutton‐Brock & Pemberton, 2004). In 1932, just over 100 Soay sheep were moved to the larger island of Hirta after the evacuation of all human residents and livestock. The population expanded and now fluctuates between 600 and 2200 sheep. Approximately one‐third of the Hirta population lives in the Village Bay area, and these individuals have been the subject of a long‐term study since 1985 (Clutton‐Brock & Pemberton, 2004). In April, around 95% of all lambs born in this area are caught each year and individually tagged; most females have a single lamb per year, with twins occurring in ~13% of births. Soay sheep are sexually dimorphic in body size, with adult males generally around 1.5 times heavier than females. They also differ in their coat colour (light/dark), a categorical trait that is determined visually at birth and does not change over the lifetime of the sheep. Each August, as many sheep as possible from the study population are recaptured using temporary traps (Clutton‐Brock & Pemberton, 2004). At capture, animals are weighed, and blood samples are collected. Whole blood samples are collected into heparin tubes, centrifuged at 3000 r.p.m. for 10 min, and plasma removed and stored at −20°C. First‐winter mortality can be high, but individuals that survive to maturity can be long‐lived (up to 16 years for females with a median of 5 years; and up to 11 years for males with a median of 2 years) (Clutton‐Brock & Pemberton, 2004). This analysis included all animals that were caught and blood sampled in August between 2011 and 2016, comprising 1452 samples from 917 individuals. A breakdown of the data set by sex, age group and year is shown in Table S1.

### 2.2 Vitamin D measurements

In the Soay sheep population on Hirta, vitamin D_2_ is available from dietary sources and vitamin D_3_ is only obtained from cutaneous production (Clutton‐Brock & Pemberton, 2004; Jäpelt & Jakobsen, 2013). This population of Soay sheep do not receive any diet supplementation, and therefore we are confident that vitamin D_2_ was obtained via grazing, whereas vitamin D_3_ was only obtained from cutaneous production. We therefore predict that individual variation in foraging ability alongside differential access to vitamin D_2_‐rich vegetation will be important regulators of vitamin D_2_ status. In terms of vitamin D_3_, we predict that access to ultraviolet B (UVB) radiation will be the critical regulator and so behavioural differences resulting in differential exposure to UVB are likely to drive some of the variation we see in vitamin D_3_ concentrations. Vitamin D is initially metabolized to 25(OH)D in the liver before undergoing a second hydroxylation in the kidney to the metabolically active 1,25 dihydroxyvitamin D (1,25(OH)_2_D). Circulating concentrations of 25(OH)D are widely used to assess vitamin D status in mammals (Elder & Bishop, 2014). Plasma concentrations of 25(OH)D_2_ and 25(OH)D_3_ were determined by liquid chromatography tandem mass spectrophotometry (LC‐MS/MS) using an ABSciex 5500 tandem mass spectrophotometer and the Chromsystems 25OHD kit for LC‐MS/MS following the manufacturers’ instructions (intra‐ and interassay coefficient of variation 3.7% and 4.8% respectively) in a laboratory certified as proficient by the international Vitamin D Quality Assurance Scheme (DEQAS). Total 25(OH)D was defined as the sum of 25(OH)D_2_ and 25(OH)D_3_ (hereafter total vD, vD_2_ and vD_3_ respectively).

### 2.3 Phenotypic associations with vitamin D concentrations

We first investigated phenotypic associations with total vD, vD_2_ and vD_3_ concentrations using a linear mixed‐effects model in the ime4 package version 1.1‐21 (Bates et al., 2015) in r version 3.5.3. We included sex (factor), age (years, linear and quadratic), coat colour (factor: light/dark), weight (linear) and year (factor) as fixed effects and individual identity and birth year as random effects. We included these fixed effects in our models because associations between sex, age and body mass index (BMI) have previously been documented in human populations (Cai et al., 2020; Muscogiuri et al., 2019; Pereira‐Santos et al., 2015; Perry et al., 1999; Zakharova et al., 2019). We predicted declines in vD_2_ but not vD_3_ concentrations with age, as foraging ability may decline with age, but sheep are unlikely to modify their UV exposure. We also predicted heavier sheep would have higher vD_2_ concentrations, since this suggests these sheep are better foragers. Further, associations with total vD and vD_3_ concentrations and coat colour have previously been documented in other ruminants (Callaby et al., 2020; Zhou et al., 2019) and so we expected sheep with dark coats to have lower concentrations of these vitamin D metabolites. Although solar radiation is a considerable driver of vitamin D status in human populations (Holick, 2007), we only had data for 6 years in this population; individual exposure levels were highly similar between all sheep measured within a given year due to the small range of sampling dates in August. Therefore, we included year to account for these and other types of environmental differences in this population. In a subset of adult female sheep (aged ≥1 year), we investigated whether there were differences between vitamin D concentrations in August for females that did not have a lamb(s), females that had a lamb(s) of which any survived to weaning (measured as whether they had any lambs surviving to 3 months old) or females that had a lamb(s) of which none survived to weaning. This was included as a three‐level factor in the model and post hoc testing was carried out using emmeans version 1.6.1 (Lenth, 2011). Although vitamin D concentrations have been shown to be negatively associated with lactation stage in dairy cattle (Holcombe et al., 2018), we expected more modest associations between previous reproductive outcomes and vitamin D status in sheep (Goyal et al., 2016). These models had the same model structure as above, with the exception that birth year was dropped from the model of vD_2_ due to lack of model convergence. The significance of fixed effects was determined by dropping each fixed effect from a model containing all terms and performing a likelihood ratio test on the two models refitted with maximum likelihood.

### 2.4 SNP and pedigree data set

A total of 7832 Soay sheep have been genotyped at 51,135 single nucleotide polymorphism (SNP) markers on the Illumina Ovine SNP50 BeadChip. Quality control was carried out using the *check.marker* function in genabel version 1.8‐0 (Aulchenko et al., 2007) in r version 3.4.3 using the following thresholds: SNP minor allele frequency (MAF) >0.001, SNP locus genotyping success >0.99, individual sheep genotyping success >0.95; identity by state with another individual <0.9. Following quality control, 39,398 SNPs and 7700 IDs remained. A pedigree has previously been inferred using 438 unlinked SNPs in the r package sequoia (Huisman, 2017); in cases where SNP information was not available, then parentage was inferred from field observations (for mothers) or from microsatellite data (Morrissey et al., 2012). We genotyped 189 sheep at 606,066 SNP loci on the Illumina Ovine Infinium HD SNP BeadChip; these sheep were selected to maximize the genetic diversity represented in the full population to allow for genotype imputation (for full details see Johnston et al., 2016). A total of 430,702 SNPs for 188 individuals passed quality control using the thresholds above. Full details of the SNP imputation are given in Stoffel et al. (2021). Briefly, SNP genotypes from the HD chip were imputed into the 50K typed individuals using alphaimpute version 1.98 (Antolín et al., 2017; Hickey et al., 2012), resulting in a data set with 7,691 individuals, 420,556 SNPs, a mean genotyping rate per individual of 99.5% (range 94.8%–100%) and a median imputation accuracy of 99.3% (assessed using a 10‐fold leave‐one‐out cross‐validation approach). All genotype positions from both SNP chips were based on the Oar_v3.1 sheep genome assembly (GenBank assembly ID GCA_000298735.1) (Jiang et al., 2014). A total of 900 out of 917 unique individuals with vitamin D measures had corresponding genomic data.

### 2.5 Animal models

We fitted quantitative genetic “animal models” (Henderson, 1975; Kruuk, 2004) in order to determine the heritability and repeatability of total vD, vD_2_ and vD_3_ concentrations in asreml‐r 4.1.0 (Butler et al., 2009) in r version 3.6.2. A genomic relatedness matrix (GRM) at all polymorphic autosomal markers on the SNP50 BeadChip was constructed for all 7700 genotyped individuals using gcta version 1.91.1 (Yang et al., 2011) with the argument ‐‐*grm*‐*adj 0*, which assumes that causal and genotyped loci have similar frequency spectra. Univariate animal models were fitted for each of the three vitamin D measures across all ages. The fixed effect structure for the model included sex and year of capture as factors, and age as a linear and quadratic covariate. We previously included coat colour and weight as a fixed effect in the model of phenotypic associations with vitamin D concentrations. However, including these in the animal models could underestimate the additive genetic variance underlying vitamin D concentrations, given that weight and coat colour themselves have an additive genetic component (Wilson, 2008). The random effects included the additive genetic component (using the GRM), permanent environment component (due to repeated measures within an individual), maternal identity and birth year. The significance of random effects was determined by dropping each random effect from a model containing all random effects and performing a likelihood ratio test distributed as $\chi_{1}^{2}$. The proportion of the phenotypic variance explained by each random effect was estimated as the ratio of the relevant variance component to the total phenotypic variance. The heritability of each measure was determined as the ratio of the additive genetic variance to the total phenotypic variance. The repeatability (i.e., the between‐individual variation) of each measure was determined as the ratio of the sum of the additive genetic and permanent environmental variance to the total phenotypic variance (equivalent to the proportion of variance explained by individual identity).

Bivariate animal models were fitted with vD_2_ and vD_3_ concentrations as the two response variables to estimate covariances at the additive genetic (using the GRM), permanent environment and residual levels. Fixed effects were fitted as above. Covariance components were summed to estimate the total phenotypic covariance between each pair of traits. Correlations were determined by dividing the covariance by the product of the square roots of the two variances at the additive genetic, permanent environment and residual levels. The significance of the genetic correlation was determined by running the bivariate model and using the *corgh* function to fix the genetic correlation at 0 or 1 (0.999) and comparing with the observed model using a likelihood ratio test.

### 2.6 Genome‐wide association study

A GWAS (Hirschhorn & Daly, 2005) using the imputed SNP data set (*N*_SNPS_ = 430,702) was used to determine associations between individual SNPs and the three vitamin D measures using the r package repeatabel version 1.1 (Rönnegård et al., 2016) in r version 3.4.3. First, a linear mixed‐effect model was constructed using the same model structures as for the animal models above using the function *preFitModel*. Then, the function *rGLS* was used to fit SNP effects in the model. This approach allowed us to fit multiple measures per individual and the GRM, which accounted for the effects of pseudoreplication and population structure, respectively (Rönnegård et al., 2016). The association *p*‐values were corrected for any additional unaccounted population structure by dividing them by the genomic control parameter *λ*, in cases where *λ* > 1, to reduce the incidence of false positives. The threshold for genome‐wide significance was previously determined using linkage disequilibrium (LD) information to determine the effective number of independent tests (Stoffel et al., 2021); the genome‐wide significance threshold was set at *p* < 1.28 × 10^−6^ at *α* =.05. In genome regions where an SNP locus was significantly associated with vitamin D, we first estimated the genotype effect sizes by fitting the genotype as a three‐level factor in the animal model structure above. Then, in separate models, the proportion of phenotypic variance explained by that region was modelled as follows: a second “regional” GRM was constructed using 10 flanking loci from the SNP50 BeadChip on either side (i.e., at least 20 SNPs in total) or the first/last 20 50K‐SNPs of the chromosome if the number of flanking SNPs on one side was <10; this regional GRM was then fitted as an additional random effect in the animal model above and significance was determined using a likelihood ratio test as above.

To identify potential candidate genes for vitamin D measures in significant regions, sheep gene IDs and their associated gene ontology (GO annotations) within 1 Mb of the most highly associated SNPs (*p* < 10^−5^) were extracted from Ensembl (gene build ID Oar_v3.1.100) using the function *getBM* in the r package biomart version 2.34.2 (Durinck et al., 2009). This threshold was selected as there is extensive LD within the Soay sheep system, with the *R* ^2^ value of LD remaining at around 0.2 at 1 Mb (Bérénos et al., 2014). Gene orthologues in humans (*Homo sapiens*), cattle (*Bos taurus*), mouse (*Mus musculus*) and rat (*Rattus norvegicus*) and associated GO terms were also extracted using the function *getLDS*. Gene and orthologue names, descriptions, phenotype descriptions and GO terms were then queried for terms associated with 25(OH)D concentrations in human studies (Jiang et al., 2018), using the r command *grep* with the strings *vitamin D*, *cholecalcif* (vitamin D_2_), *ergoecalcif* (vitamin D_3_), *CYP2*, *NADSYN1*, *DHCR7*, *SEC23A* and *AMDHD1*.

### 2.7 Fitness analyses

We investigated associations between total vD, vD_2_ and vD_3_ concentrations measured in summer and survival over the subsequent winter, since the vast majority of sheep deaths occur over the winter months (Clutton‐Brock & Pemberton, 2004). Accurate death date information is known from regular censuses and searches for carcasses over this period. Over‐winter survival was defined as survival from capture and sampling in August to May 1 in the subsequent year. Due to known differences in survival rates between lambs and adult Soay sheep, we modelled lambs and adults separately (where the adult group included all sheep aged ≥1 year). Both lamb and adult survival analyses were conducted using generalized linear models (GLMs) with a binomial error distribution and a logit link function. The lamb base model included sex (factor), coat colour (factor: light/dark), weight (covariate) and year (factor) as fixed effects. The base model for adults included the fixed effects of sex (factor), age group (factor with three levels: yearlings, 2–6 years and ≥7 years), coat colour (factor), weight (covariate) and year (factor) and a sex by age group interaction to account for differences between the sexes in survival probability for each age group. Previously we included age as a quadratic term in the models with vitamin D concentrations as the response variable. Here, in the models of survival and breeding success we included age group to try and model the curvature of the age relationship with survival and breeding success while also allowing interactions with age to be more interpretable. Covariates were rescaled to mean 0 and standard deviation 1 prior to inclusion in each model. To investigate associations between vitamin D status and over‐winter survival, we compared the Akaike information criterion (AIC) of this base model with models containing total vD, vD_2_ and vD_3_ separately and a model with both vD_2_ and vD_3_ fitted additively. We also ran these models with interactions between each vitamin D measure and sex, year and age group (adult models only) to test for environment‐, sex‐ and age‐dependent associations between vitamin D measures and survival or breeding success. The model with the lowest AIC value was considered the best‐fitting model, unless the difference in AIC was within 2 AIC units of a model with fewer explanatory terms, in which case this simpler model was considered the most parsimonious (Burnham & Anderson, 2002). We chose a model selection approach rather than including all vitamin D measures in one model or model averaging, as total vD is the sum of vD_2_ and vD_3_ and there were moderate to high correlations between the measures (Pearson's product‐moment correlations: vD_2_ & vD_3_ *r* = .59, *t*_1450_ = 28.03, *p* < .001; total vD & vD_2_ *r* = .73, *t*_1450_ = 40.80, *p* < .001; total vD & vD_3_ *r* = .98, *t*_1450_ = 202.98, *p* < .001). For each chosen model in the results we provide Akaike weights and Nagelkerke pseudo *R* ^2^ (Nagelkerke, 1991) or conditional *R* ^2^ values (Nakagawa et al., 2017) for GLMs and generalized linear mixed models respectively.

Next, we tested for associations between total vD, vD_2_ and vD_3_ concentrations and breeding success in the following spring. For females that survived the winter, annual breeding success was defined as the number of offspring born to the female in the following spring derived from observational data. For males, annual breeding success was calculated as the number of offspring sired in the following spring using the genetic pedigree. Male annual breeding success included all males that died over the winter but excluded all males that were not observed to take part in the rut using census records taken between October and December. Since the distribution of annual breeding success is very different between males and females, and between lambs and adults, models were run for female lambs, male lambs, adult females and adult males separately (where the adult group included all sheep aged ≥1 year). For female lambs, breeding success was treated as a binary measure (produced a lamb/did not produce a lamb) as no females had twins in their first year and the model was run as a GLM with a binomial error distribution and a logit link function. In this model, fixed effects included coat colour (factor), weight (covariate) and year (factor). Data from 2011 were excluded due to the small number of available measures (*n* = 4) driven by very high lamb over‐winter mortality, but the results were consistent with or without these measures (Table S2). For adult females, which have zero to two lambs per year, annual breeding success analyses were conducted using a proportional odds mixed model in the ordinal package version 2019.12‐10 using the adaptive Gauss–Hermite quadrature approximation (Christensen, 2018). Fixed effects included age group (factor, levels as before), coat colour (factor), weight (covariate) and year (factor) with individual identity included as a random effect. Since the probability of males siring offspring in their first year is low (10% in this data set), male lamb breeding success was treated as a binary measure (sired a lamb/did not sire a lamb) and the model was run as a GLM with a binomial error distribution and a logit link function with coat colour (factor), weight and year (factor) as fixed effects. No males born in 2011 in this data set sired offspring in their first year so this year was excluded from analyses to improve model convergence, although results were consistent with or without these measures (Table S2). Finally, for adult males we analysed annual breeding success in the glmmtmb package version 1.0.1 (Brooks et al., 2017) where the best‐fitting model was the negative binomial model with the “nbinom1” parametrization without zero inflation. Fixed effects in this model included age group (factor), coat colour (factor), weight and year (factor) with individual identity as a random effect. Continuous variables were rescaled to mean 0 and standard deviation 1 prior to inclusion in each model. As for the survival analyses, AIC values of base models were compared with models with total vD, vD_2_, vD_3_ separately and vD_2_ and vD_3_ combined and models where there was a vitamin D measure by year or age group (adult models only) interaction.

## 3 RESULTS

### 3.1 Individual variation in vitamin D concentrations

Vitamin D_2_ and D_3_ concentrations were positively correlated with one another (Pearson's product‐moment correlation: *r* = .59, *t*_1450_ = 28.03, *p* < .001; Figure S1) but vD_3_ concentrations contributed more to the total vD concentrations and were on average 26.13 nmol L^–1^ higher (paired *t* test: 95% confidence interval [CI] 25.04–27.23 nmol L^–1^) than vD_2_ concentrations. There was a quadratic association with all vitamin D measures and age (Tables S3 and S4, Figures S2 and S3). In models where age was fitted as a factor, there was a substantial increase in concentrations of the vitamin D metabolites between lambs (Age 0) and yearlings (Age 1) of 33.79 nmol L^–1^ (± 1.76 *SE*), 4.50 nmol L^–1^ (± 0.46 *SE*) and 29.30 nmol L^–1^ (± 1.52 *SE*) for total vD, vD_2_ and vD_3_ respectively. For vD_2_, this was followed by a decline with age that was not as apparent for total vD and vD_3_ (Tables S3 and S4, Figure S2). After accounting for weight in the model, there was a difference in total vD, vD_2_ and vD_3_ concentrations between the sexes, with males having lower concentrations of all three measures compared to females (Table S3; without weight: total vD *β* = −2.142 ± 1.225 *SE*, *χ* ^2^_(1)_ = 3.123, *p* = .077; vD_2_ *β* = −0.186 ± 0.319 *SE*, *χ* ^2^_(1)_ = 0.359, *p* =.549 and vD_3_ *β* = −1.995 ± 1.047 *SE*, *χ* ^2^_(1)_ = 3.699, *p* = .054). Sheep with light coats had significantly higher total vD and vD_3_ concentrations of 5.81 nmol L^–1^ (±1.33 *SE*) and 6.46 nmol L^–1^ (±1.15 *SE*) respectively compared to sheep with dark coats (Table S3). Light coated sheep had slightly lower vD_2_ concentrations of −0.67 nmol L^–1^ (±0.34 *SE*) compared to dark coated sheep although this association was only marginally significant (Table S3, *p* = .048). There was a positive association between weight and all vitamin D measures; heavier sheep had higher total vD, vD_2_ and vD_3_ concentrations (Table S3). All vitamin D measures varied significantly by year (Tables S3 and S4, Figure S3).

In a subset of adult female sheep, females which had a lamb that did not survive to weaning (i.e., did not survive to 3 months old) had higher total vD (estimated marginal mean [EMM] = 74.4 nmol L^–1^ ± 2.18 *SE*) and vD_3_ concentrations (EMM = 58.8 nmol L^–1^ ± 1.93 *SE*) than females which had a lamb that survived to weaning (total vD EMM = 69.4 nmol L^–1^ ± 1.59 *SE*; vD_3_ EMM = 54.2 nmol L^–1^ ± 1.43 *SE*) or those that did not have a lamb that spring (total vD EMM = 66.6 nmol L^–1^ ± 2.33 *SE*; vD_3_ EMM = 52.0 nmol L^–1^ ± 2.06 *SE*; Tables S5 and S6). However, there was no significant difference in total vD or vD_3_ concentrations between females which did not have a lamb and those which had a lamb that survived to weaning (Table S6). There was no significant difference in vD_2_ concentrations between these three adult female groups (Table S5).

**FIGURE 1:**
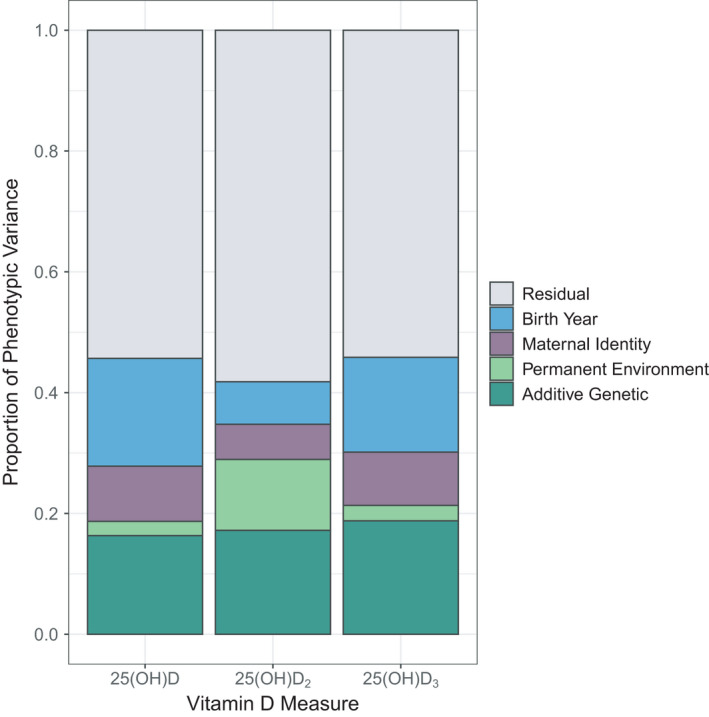
The proportion of phenotypic variance explained by different random effects in univariate animal models for total vD (25(OH)D), vD_2_ (25(OH)D_2_) and vD_3_ (25(OH)D_3_) plasma concentrations in wild Soay sheep

There was considerable among‐individual variation in each of the three vitamin D measures, with repeatabilities of 0.19 for total vD, 0.29 for vD_2_ and 0.28 for vD_3_ (Table S7; Figure 1), where most of this variation (59%–87%) was explained by the additive genetic component (see next section). The consistency of vitamin D concentrations within individuals is further illustrated by the strong positive correlation between measures taken from the same individual across consecutive years in adults (Figure S4; Pearson's product‐moment correlations: total vD: *r* = .62, *t*_350_ = 14.89, *p* < .001; vD_2_: *r* = .76, *t*_350_ = 21.82, *p* < .001; vD_3_: *r* = .58, *t*_350_ = 13.21, *p* < .001).

### 3.2 Genetic architecture of vitamin D concentrations

**FIGURE 2:**
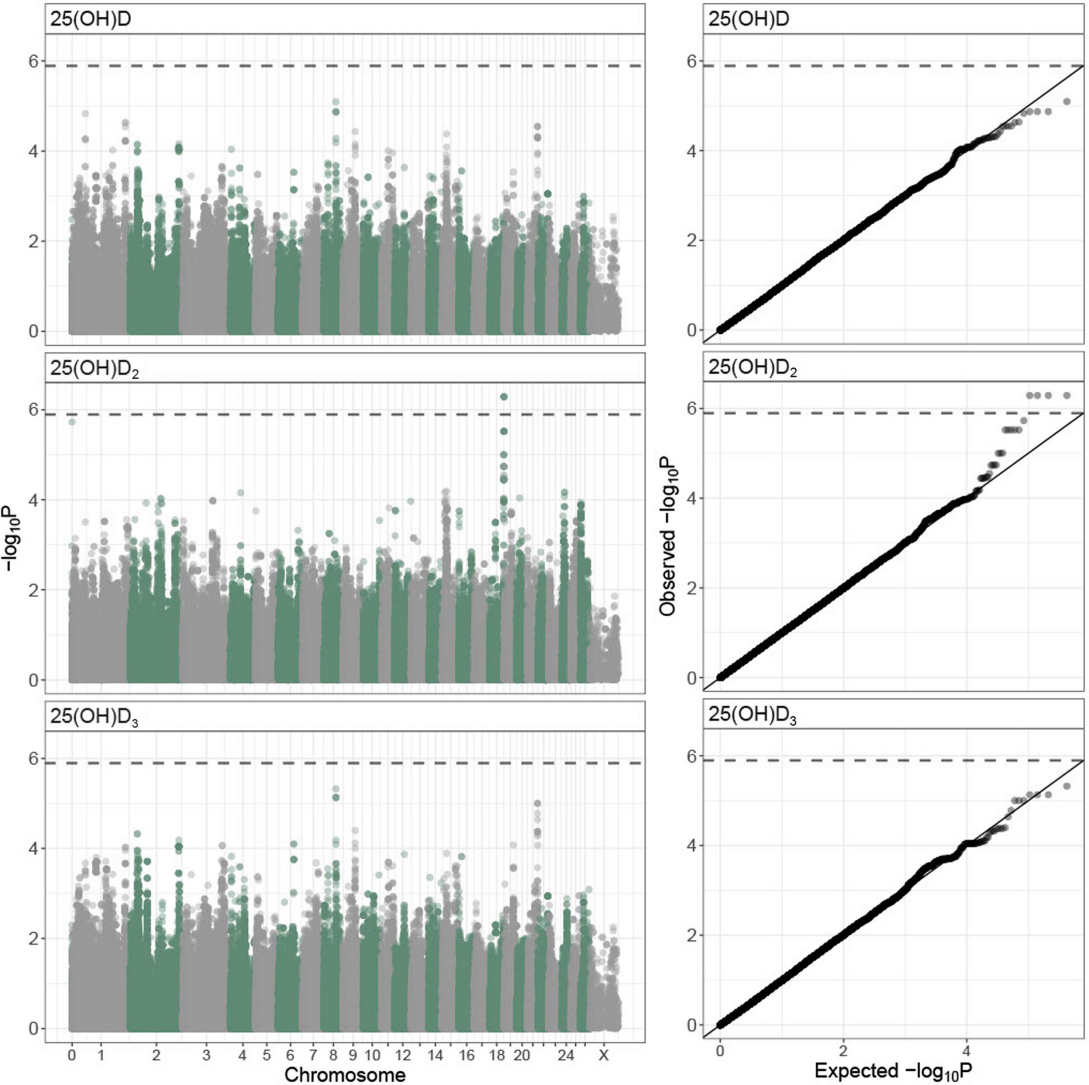
Genome‐wide association results for total vD (25(OH)D), vD_2_ (25(OH)D_2_) and vD_3_ (25(OH)D_3_) concentrations at 420,556 SNPs in 900 Soay sheep across all ages. Left panel: Manhattan plots of *p*‐values are corrected for inflation (by dividing by *λ* – total vD: 2.16, vD_2_: 1.94, vD_3_: 2.14) and the dashed line indicates the genome‐wide significance threshold. Right panel: *p–p* plots showing the association between expected and observed (*λ* corrected) *p*‐values where the solid line indicates the one to one line and the dashed line indicates the significance threshold

All three vitamin D measures were heritable: *h* ^2^ = 0.16 (± 0.04 *SE*) for total vD, *h* ^2^ = 0.17 (±0.04 *SE*) for vD_2_ and *h* ^2^ = 0.19 (±0.04 *SE*) for vD_3_ (Table S7; Figure 1). Variance attributable to birth year ranged from 7.1% to 17.8% across vitamin D measures and there was evidence for maternal effects for total vD and vD_3_ measures (2.5%–9.1% of the total phenotypic variance; see Figure 1; Table S7). A bivariate model of vD_2_ and vD_3_ showed there was a moderate positive additive genetic correlation between the two traits (*r*_a_ = .32 ± .13 *SE*; Table S8). This correlation was significantly different from 0 (*χ* ^2^_(1)_ = 24.18, *p* < .001) and 1 (*χ* ^2^_(1)_ = 73.17, *p* < .001). A GWAS found four SNPs in a single region at the distal end of chromosome 18 (68,320,039 to 68,448,747 bp; SNPs *oar3_OAR18_68320039*, *oar3_OAR18_68398710*, *oar3_OAR18_68401733* and *oar3_OAR18_68448747*) were significantly associated with vD_2_ concentrations (MAF = 0.219, *χ* ^2^_(1)_ = 50.08, *λ*‐corrected *p* = 5.16 × 10^−7^; Figure 2; Figure S5, Table S9). All four SNPs were in very strong LD with one another (*R* ^2^ > .99) and had equivalent effects on phenotype. An animal model with genotype at *oar3_OAR18_68320039* fitted as an additional fixed factor found that genotypes AC and CC increased vD_2_ concentrations by 0.91 (±0.72 *SE*) and 2.40 (±0.73 *SE*) nmol L^–1^, respectively (Wald *p* = 2.12 × 10^−6^; note the AA and AC genotypes were not significantly different). This window contained three unannotated protein‐coding regions, orthologous to the genes *TEX22*, *TEDC1* and *TMEM121*. None of these genes have previously been associated with vitamin D; similarly, there were no further candidate genes/orthologues within 1 Mb of this association. Inclusion of a regional GRM in the animal model for vD_2_ significantly improved the model (*χ* ^2^_(1)_ = 10.31, *p* = .001), showing that the chromosome 18 association region explained 6.56% (*SE* = 4.10%) of the phenotypic variance and 14.44% (*SE* = 4.06) of the additive genetic variance underlying vD_2_ concentrations. No SNPs were associated with total vD or vD_3_ concentrations at the genome‐wide level.

### 3.3 Associations between vitamin D concentrations and individual fitness

**FIGURE 3:**
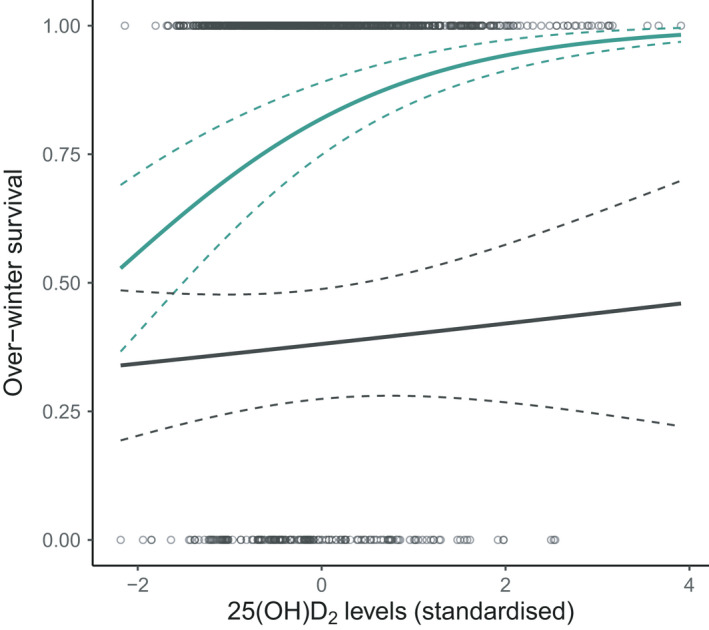
Scatterplot of raw data and general linear model predictions for associations between vD_2_ (25(OH)D_2_) concentrations (standardized, for full details see Section 2) and subsequent over‐winter survival probability for adult female (green) and male (grey) Soay sheep. The slope is predicted from the model with both sexes (Table S10) with other fixed effects set as follows: yearling age group, dark coat colour, the year 2011 and average weight. The dashed lines represent standard errors

There was no strong evidence of an association between vitamin D measures in lambs and their subsequent first‐winter survival (Table 1, chosen model: Nagelkerke pseudo‐*R* ^2^ = .397, model weight = 0.10). However, in adults, there was a significant interaction between vD_2_ and sex on over‐winter survival (*χ* ^2^_(1)_ = 4.790, *p* = .029, Table 1; Table S10; Figure 3, chosen model: Nagelkerke pseudo‐*R* ^2^ = .386, model weight = 0.55), in which females with higher vD_2_ concentrations in August were more likely to survive to the following year independently of their age and weight (females‐only model: *b* = 0.607 ± 0.182 *SE*, *χ* ^2^_(1)_ = 12.119, *p* < .001, Table S10). However, there was no significant association between vD_2_ concentrations and winter survival for adult males (males‐only model: *b* = 0.177 ± 0.246, *χ* ^2^_(1)_ = 0.522, *p* = .470, Table S10).

**TABLE 1:** AIC comparison of over‐winter survival and annual breeding success models in Soay sheep

	AIC comparisons to best‐fitting model					
	Winter survival		Female annual breeding success		Male annual breeding success	
	Lambs (n_IDs_ = 520)	Adults (n_obs_ = 851, n_IDs_ = 415)	Lambs (n_IDs_ = 108)	Adults (n_obs_ = 578, n_IDs_ = 245)	Lambs (n_IDs_ = 213)	Adults (n_obs_ = 181, n_IDs_ = 109)
Base model	**0.99**	10.74	**1.81**	10.83	***0.00***	***0.00***
Total vD	*0.00*	6.98	0.74	9.30	1.82	1.22
vD_2_	0.74	2.79	3.60	11.75	1.50	0.97
vD_3_	0.80	9.12	*0.00*	9.51	1.95	1.49
vD_2_ + vD_3_	1.74	4.50	1.93	11.31	3.50	2.88
Total vD × Sex	0.93	8.70	—	—	—	—
vD_2_ × Sex	1.93	***0.00***	—	—	—	—
vD_3_ × Sex	2.05	11.12	—	—	—	—
vD_2_ × Sex + vD_3_ × Sex	4.55	3.30	—	—	—	—
Total vD × Age group	—	6.94	—	9.62	—	4.85
vD_2_ × Age group	—	6.51	—	15.04	—	4.92
vD_3_ × Age group	—	8.61	—	9.78	—	5.01
vD_2_ × Age group + vD_3_ × Age group	—	8.17	—	15.44	—	10.07
Total vD × Year	9.41	16.52	5.16	***0.00***	4.90	2.06
vD_2_ × Year	5.77	11.75	7.84	8.57	5.47	4.40
vD_3_ × Year	9.43	18.45	5.60	0.12	3.67	2.27
vD_2_ × Year + vD_3_ × Year	13.95	22.27	5.61	2.84	8.91	11.58

Note

The best‐fitting model (where ΔAIC = 0) is highlighted in italics, and the most parsimonious model is highlighted in bold where ΔAIC < 2 to the best‐fitting model. All models with interactions also include these variables separately as main effects. Sample sizes are given by the number of measurements (n_obs_) and the number of unique individuals (n_IDs_) for each model.

**FIGURE 4:**
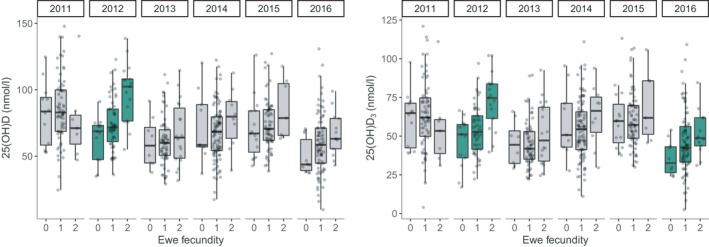
Boxplots of raw data showing associations between total vD (25(OH)D) and vD_3_ (25(OH)D_3_) plasma concentrations and adult female fecundity (number of lambs born) by year in Soay sheep. Green boxplots indicate years in which there was a significant association (*p* < .05, Table S12) between total vD or vD_3_ and female fecundity

In females, there was no strong evidence of an association between vitamin D measures of lambs and the probability of breeding in their first year (Table 1, chosen model: Nagelkerke pseudo‐*R* ^2^ = .418, model weight = 0.11). However, in adult females, the best fitting models included a significant interaction between total vD and vD_3_ and year on annual breeding success (total vD × Year: *χ* ^2^_(5)_ = 19.304, *p* = .002, Nakagawa conditional *R* ^2^ = .687, model weight = 0.44; vD_3_ × Year: *χ* ^2^_(5)_ = 19.389, *p* = .002, Nakagawa conditional *R* ^2^ = .687, model weight = 0.42, Table 1; Table S11; Figure 4). Total vD and vD_3_ concentrations in August positively predicted a female's fecundity the following spring, but only in certain years (Tables S11 and S12; Figure 4). In 2012, both total vD and vD_3_ concentrations positively predicted a female's fecundity the following spring and in 2016 there was a marginally significant association between vD_3_ concentrations and female fecundity (Table S12; Figure 4). For males, there was no strong evidence for associations between any of the vitamin D measures and the probability of males siring offspring in their first year (Table 1; chosen model: Nagelkerke pseudo‐*R* ^2^ = .204, model weight = 0.27), or annual breeding success in subsequent years for adult males (Table 1; chosen model: Nakagawa conditional *R* ^2^ = 0.778; model weight = 0.21).

## 4 DISCUSSION

This study investigated the genetic architecture underlying vitamin D status in a wild mammal population and associations with fitness under natural conditions. We demonstrate that vitamin D status is repeatable within the lifetime of individuals and is moderately heritable. We found limited evidence that heritable variation in vitamin D status was underpinned by loci of major phenotypic effect: a single genomic region explained ~6.6% of the phenotypic variance in vD_2_, but almost all heritable variation in circulating total vD, vD_2_ and vD_3_ concentrations appears to be driven by many loci of small effect. Further, we have shown that vitamin D status is under age‐, sex‐ and environment‐dependent selection and that selection patterns differed between cutaneous (vD_3_) and orally derived sources (vD_2_) of vitamin D. Diet‐derived vitamin D metabolite concentrations (vD_2_) were positively associated with female adult survival across all years whereas cutaneously produced concentrations (vD_3_) best predicted female fecundity in certain good environment years when sheep density and competition for resources was low (Figure S6). Our study provides evidence that vitamin D status is heritable and has the potential to respond to selection under natural conditions, which offers a unique insight into how vitamin D metabolism is associated with fitness in the wild.

We found considerable nongenetic sources of variation in serum vitamin D concentrations in this population. First, previous longitudinal studies in humans have documented declines in serum total vD with age, which have been implicated with reduced sun exposure (Perry et al., 1999). In our Soay sheep population, we found a pronounced decline in dietary‐derived vD_2_ with age which is likely to indicate age‐related declines in foraging or foraging efficiency. In support of this, we have previously observed within‐individual declines in home range area with age in female Soay sheep (Froy et al., 2018). Second, sex differences in vitamin D status have been reported in some studies in humans, with females having lower total vD concentrations (Cai et al., 2020; Muscogiuri et al., 2019). We found that Soay sheep females had higher vitamin D concentrations, but only after accounting for weight. A study in cattle found no association between sex and total vD concentrations (Callaby et al., 2020), suggesting that the sex differences in humans may be due to differences in behaviour and diet which do not occur in domestic ruminants. Third, we found that dark coated sheep had considerably lower total vD and vD_3_, which is consistent with studies in other sheep breeds (Zhou et al., 2019) and cattle (Callaby et al., 2020). This implies that darker coats are absorbing more UVB radiation which reduces the cutaneous production of vitamin D. Fourth, while a negative association between vitamin D status and BMI has been reported in human populations (Pereira‐Santos et al., 2015; Zakharova et al., 2019), we found a positive association with weight in the Soay sheep. The explanation for this difference may relate to the fact that BMI is a measure involving both weight and height so gives a more accurate measure of body fat. In the context of wild animals, where obesity is less prevalent, weight is likely to reflect general health and foraging abilities (which is a probable driver of the positive relationship with vD_2_). The positive relationship between vD_3_ concentrations and weight may be due to variations in surface area, which is known to influence vitamin D status, particularly in settings with low UVB exposure (Bogh et al., 2011). Fifth, the strong variation in all vitamin D measures by year is likely to reflect differences in climate ahead of blood sampling in August. For example, reduced UVB radiation levels in some years may lead to lower vD_3_ production in the skin of the sheep and may similarly result in lower vD_2_ content of the vegetation. Finally, in adult females there were modest differences in total vD and vD_3_ concentrations depending on whether the female had a lamb that spring, and whether the lamb survived to weaning. This highlights the relatively modest changes in total vD which occur in sheep during the reproductive cycle (Goyal et al., 2016).

We found that all vitamin D metabolites were relatively repeatable within individuals, despite the environmental heterogeneity experienced by individuals in this population. Longitudinal studies of vitamin D status in humans have similarly found that total vD concentrations are correlated across subsequent sampling points within individuals (Jorde et al., 2010; Major et al., 2013; McKibben et al., 2016; Saliba et al., 2012; van Schoor et al., 2014). However, such studies are complicated by a more varied dietary intake in vitamin D and the greater ability of individuals to modify their UV exposure by modifying their behaviour and geographical location than in our wild mammal population. In our study, 59%–87% of the among‐individual variation (repeatability) was due to additive genetic variation. Although vitamin D status is expected to be heavily influenced by environment, namely diet for vD_2_ and sunlight for vD_3_, our data show that there is still considerable genetic variation contributing to variation in circulating concentrations of total vD, with heritabilities of 16.3%–18.8%. Previous estimates from human populations using twin or familial studies have estimated the heritability of total vD as high as 86% (Arguelles et al., 2009; Karohl et al., 2010; Mills et al., 2015; Orton et al., 2008), but more recent SNP‐based heritabilities have been modest, ranging from 7.5% to 13% (Jiang et al., 2018; Revez et al., 2020). Our results provide evidence that total vD as well as vD_2_ and vD_3_ are heritable in Soay sheep, indicating these traits have the potential to respond to selection and provide an important basis for further investigations into how genetic variation is maintained in these traits, including genotype‐by‐environment interactions.

The evolutionary potential of a given trait is also dependent on genetic correlations with other traits which may otherwise be masked at the phenotypic level (Kruuk et al., 2008). No previous studies have investigated the genetic correlation between diet‐derived vD_2_ and cutaneously produced vD_3_ concentrations, and this cannot be readily interpreted in human populations due to the widespread consumption of vitamin vD_3_‐containing foodstuffs (Schöttker et al., 2014). Our study found a moderate positive genetic correlation between the two differentially obtained metabolites of .322, suggesting that variation in plasma concentrations of vD_2_ and vD_3_ are under some degree of shared genetic control. While selection could act independently on the two metabolites, this observed genetic correlation will constrain the evolution of one of the traits if antagonistic selection were to occur. Given the differential sources of these two metabolites, there are likely to be differences in the genetic pathways or networks involved in the metabolism of these two traits. For example, diet‐derived vD_2_ concentrations could be associated with genes underlying foraging behaviour and competitive ability, and cutaneously derived vD_3_ may be related to differences in behaviour with respect to sheltering and exposure to sunlight. In contrast, vD_2_ and vD_3_ share a similar metabolism once derived from the diet and skin respectively. Both vitamin D_2_ and vitamin D_3_ are metabolized by CYP2R1 into 25(OH)D_2_ and 25(OH)D_3_ and then to 1,25(OH)_2_D_2_ and 1,25(OH)_2_D_3_ by CYP27B1 (Holick, 2007). Consequently, we would envisage that the evolutionary constraints and pressures would act similarly on both vD_2_ and vD_3_ once it has been produced. Future studies of individual variation in UV exposure and diet selection in unmanaged and unsupplemented populations such as the Soay sheep could give more insight into the natural drivers of genetic variation in vitamin D metabolism.

A GWAS identified a single genomic region on chromosome 18 explaining ~6.6% of the phenotypic variance and ~14.4% of the additive genetic variance in vD_2_. Significant SNPs on chromosome 18 did not correspond directly to any compelling candidate genes associated with total vD concentrations in humans (Ahn et al., 2010; Jiang et al., 2018; Manousaki et al., 2017; Revez et al., 2020; Wang et al., 2010). Two genes within the broader region, *PPP1R13B* and *DLK1* (~1.28 and ~2.67 Mb away, respectively), were recently implicated in a human GWAS of circulating total vD concentrations (Revez et al., 2020). There was moderate LD between SNPs corresponding to *PPP1R13B* and the most highly associated SNPs (*r* ^2^ = .765), suggesting that there may be some element of shared architecture of this trait. However, with the exception of this association, almost all heritable variation in vitamin D concentrations appears to have a polygenic basis (i.e., variation is driven by many genes of small effect throughout the genome). The high levels of LD in the Soay sheep genome aid the detection of trait loci using GWAS approaches. However, our sample sizes are considerably lower than those of human studies (Manousaki et al., 2020; Revez et al., 2020), which may reduce the power to detect associations of moderate effect sizes and/or at alleles of low frequency within the population. Nevertheless, previous GWAS of body size, antibody levels and recombination rate in Soay sheep using only one tenth of the current SNP marker data set successfully identified multiple loci explaining 2.6%–46.7% of the phenotypic variance in these traits (Bérénos et al., 2015; Johnston et al., 2016; Sparks et al., 2019). Consequently, it is unlikely that there are major effect loci underpinning total vD, vD_2_ or vD_3_ plasma concentrations in the Soay sheep. A previous study in this system documented that ewes with a light coat had higher total vD and vD_3_ concentrations than ewes with a dark coat (Handel et al., 2016) and this association was confirmed in both sexes in this 6‐year data set. The light/dark coat colour polymorphism is determined by a single base pair substitution at the *TYRP1* locus on chromosome 2 (Gratten et al., 2007). However, this region was not associated with vitamin D status in the GWAS, suggesting that coat colour in itself does not explain enough variation in vitamin D status in this population for this region to have been detected.

In this study, we were able to test for associations between vitamin D status and mortality for the first time in a wild population, and were able to dissect associations between total vD, oral (vD_2_) and cutaneously derived (vD_3_) sources of vitamin D. Vitamin D_2_ and D_3_ differ structurally and there is increasing evidence from human clinical trials that vD_2_ is not as effective as vD_3_ in increasing total vD concentrations (Martineau et al., 2019). However, the clinical relevancy of this difference remains ill‐defined in humans. Previous studies in both human and companion animals have demonstrated an association between low total vD concentrations and poor health outcomes, including mortality (Jaffey et al., 2018; Schöttker et al., 2014; Titmarsh, Gow, et al., 2015; Titmarsh, Kilpatrick, et al., 2015). Interestingly, in this population we found a positive association between diet‐derived vD_2_ and over‐winter survival in adult female sheep, but no association between total vD or vD_3_ concentrations during August and subsequent over‐winter survival. This association between vD_2_, but not total vD or vD_3_ summer concentrations, and female over‐winter survival could be indicative of the environment experienced by the study population: no individuals receive any vitamin D_3_‐supplemented food over winter and individuals live at a latitude where cutaneous production of vitamin D_3_ is not possible between November and March, which is the time period where mortality is highest on the island (Clutton‐Brock & Pemberton, 2004; Hurst, Homer, et al., 2020; Smith & Wright, 1984). Consequently, vD_2_ may constitute almost all of the total winter vD concentration and so it is perhaps unsurprising that vD_2_ rather than vD_3_ or total vD is linked to over‐winter survival. Prolonged low concentrations of total vD are likely to be highly deleterious to sheep, with metabolic consequences including hypocalcaemia and skeletal disorders (Dittmer & Thompson, 2011). This is particularly pertinent following the growing awareness that rickets remains a significant health issue in extensively farmed hill sheep farmed at a similar latitude to the Soay population (Hurst, Henderson, et al., 2020). This case series highlighted that cutaneously derived vD_3_ is often very low in late winter in Scottish hill sheep and so if insufficient vD_2_ is consumed during the winter period then pronounced hypovitaminosis D with associated metabolic and skeletal complications can develop (Hurst, Henderson, et al., 2020).

The lack of an association between summer vitamin D status and over‐winter survival in males may be due to the smaller sample size of males in the study or because males rut before the winter and forgo grazing (Clutton‐Brock & Pemberton, 2004). As a result, a summer measure of grazing ability may be unlikely to extend to the rut or reflect the status and condition of males going into the winter. Further, the absence of an association in lambs may be because they were just weaned at the time of blood sampling and were unlikely to be grazing as much as older animals, as observed by their lower plasma concentrations of vD_2_. While it is unclear whether vD_2_ is acting as a marker of foraging capability or is directly leading to ill‐health and contributing to death risk, our study provides evidence of an association between vitamin D status and subsequent mortality in a wild animal, and suggests that selection could be acting in different ways on vD_2_ and vD_3_ concentrations.

Higher plasma concentrations of vitamin D in August predicted increased fecundity in females, but not males, the following spring. This relationship was predominantly driven by vD_3_ and was highly year‐dependent; the association was observed in 2012 and was marginally significant in 2016. Previously we identified a positive association between serum total vD concentrations and female fecundity using data from 2012 only (Handel et al., 2016). Our study recapitulates this finding using plasma samples, but also suggests that this finding is only apparent under certain ecological conditions. There is considerable variation in sheep density by year in this population (Figure S6; 2011–2016 range = 362–649 sheep in the study area). This is driven by differences in winter mortality between years, which is determined by a combination of variation in density, weather and the proportion of vulnerable individuals (Coulson et al., 2001). In 2012, when the relationship between total vD and vD_3_ and ewe fecundity was highly significant, there was a low sheep density and low competition for resources during gestation and lactation following a population crash (Figure S6). The second lowest sheep density in our study period was in 2016, when there was a marginally significant association between vD_3_ and ewe fecundity. This suggests there is environment‐dependent fecundity selection for vitamin D status under benign ecological conditions. Our previous study determined that the relationship between vitamin D status and reproductive success is driven by increased fecundity rather than improved post‐parturition maternal care and lamb survival (Handel et al., 2016). The finding that increased vitamin D status is associated with improved female reproductive outcomes is consistent with findings in experimental models that vitamin D deficiency is linked to infertility, reduced pregnancy rates and litter sizes (Brommage & DeLuca, 1984; Coffey et al., 2012; Halloran & Deluca, 1980; Lerchbaum & Obermayer‐Pietsch, 2012; Marya et al., 1991; Yoshizawa et al., 1997). Conversely, the lack of an association between vitamin D status and male breeding success is unsurprising given the limited evidence that vitamin D supplementation influences male reproductive outcomes in humans (Blomberg Jensen et al., 2018; Boisen et al., 2017; Lerchbaum & Obermayer‐Pietsch, 2012). Our results provide evidence for both sex‐ and environment‐dependent selection on vitamin D status, and suggests possible mechanisms by which genetic variation may have been maintained in these traits.

In conclusion, our study provides the first insights into the genetic basis and selection on vitamin D status in a wild population. Further study is warranted to investigate how well summer vitamin D concentrations predict winter vitamin D concentrations and whether selection patterns on winter vitamin D metabolites differ. Crucially, further studies in this population are needed to establish whether there are direct effects of vitamin D status on mortality and fecundity, or whether these measures are picking up signals of correlated traits such as diet choice, foraging capability or other aspects of behaviour that are themselves associated with fitness.

## AUTHOR CONTRIBUTIONS

This study was conceived by A.M.S., R.J.M. and D.H.N. J.M.P., J.G.P. and D.H.N. manage the long‐term Soay sheep study system. Samples were collected by J.M.P. and J.G.P. Laboratory work was undertaken by J.B. A.M.S. and S.E.J. performed all data analyses with inputs from D.H.N., I.H. and R.J.M. A.M.S. wrote the first draft of the manuscript with input from S.E.J., R.J.M. and D.H.N. All authors provided comments on the manuscript and gave final approval for publication.

## Supporting information

Supplementary Material

Table S9

## Data Availability

Data are available from the Dryad Digital Repository: https://doi.org/10.5061/dryad.w0vt4b8sq (Sparks et al., 2021). All scripts for the analysis are provided at https://github.com/sejlab/Soay_Vitamin_D.
